# Effects of Rumen-Protected Methionine and Lysine on the Fecal Microbiota of Leizhou Goats

**DOI:** 10.3390/microorganisms13112433

**Published:** 2025-10-23

**Authors:** Weishi Peng, Hu Liu, Ke Wang, Yuanting Yang, Anmiao Chen, Meng Zeng, Qun Wu, Jiancheng Han, Mao Li, Hanlin Zhou

**Affiliations:** 1 Zhanjiang Experimental Station, Chinese Academy of Tropical Agricultural Sciences, Zhanjiang 524013, China; pweishi@126.com (W.P.); liuh2018@lzu.edu.cn (H.L.); lp_wangke@163.com (K.W.); ytyang10@163.com (Y.Y.); cam1835287831@163.com (A.C.); zmeng0909@163.com (M.Z.); wuqun.2006@163.com (Q.W.); hanjiancheng810@163.com (J.H.); 2 Tropical Crops Genetic Resources Institute, Chinese Academy of Tropical Agricultural Sciences, Danzhou 571737, China

**Keywords:** rumen-protected methionine and lysine, Leizhou goats, gut microbiota, growth performance

## Abstract

This study investigates the effects of rumen-protected methionine and lysine (RPML) on the fecal microbiota of Leizhou goats, focusing on growth performance and fecal microbial community composition. A total of 10 three-month female Leizhou goats (9.90 ± 0.08 kg) were randomly assigned to one of two dietary treatments: a CON group fed a basal diet and an RPML group receiving the basal diet supplemented with 1.5 g/d/head of rumen-protected methionine and 4.5 g/d/head of rumen-protected lysine. Results indicated that RPML significantly enhanced average daily gain (ADG) and final body weight (FBW), as well as significantly decreased the ratio of dry matter intake (DMI) to ADG (*p* < 0.001). Fecal microbiota composition showed a decrease in abundance of *UCG-005*, *Phascolarctobacterium,* and *norank_f__Bacteroidales_RF16_group* and an increase in others like *Christensenellaceae R-7* and *unclassified_c__Clostridia* (*p* < 0.05). Moreover, the correlations between the abundance of certain bacterial genera and the concentrations of short-chain fatty acids (SCFAs) suggest that the modulation of the gut microbiota is associated with improved growth performance and feed efficiency in Leizhou goats, indicating that RPML supplementation can modulate the gut microbiota to improve growth performance and feed efficiency in Leizhou goats.

## 1. Introduction

Methionine and lysine are the most important limiting amino acids for ruminants, which have the functions of improving growth performance, feed conversion efficiency, and immunity [1,2]. However, under the action of complex rumen microorganisms in ruminants, methionine or lysine are degraded (degradation rate of 20~60%), which affects their efficacy. Therefore, methionine and lysine are often treated with rumen-protected technology to solve the dilemma of amino acid deficiency in production practice [3]. At present, rumen-protected methionine and lysine (RPML) have been widely used in dairy cattle, beef cattle, and sheep, but there are few research reports on Leizhou goats.

As a unique goat breed in the tropical region of southern China, the Leizhou goat is characterized by strong adaptability, high reproductive rate, and tender meat quality, and is an important part of the local livestock industry, typically raised through a combination of grazing and supplemental feeding [4,5]. This breed is typically reared in a semi-intensive system, grazing on native pastures and being supplemented with tropical forages such as king grass and cassava foliage [6,7]. This unique dietary regimen, rich in specific tropical plant resources, is known to contribute to a distinct gut microbiome structure in goats [8]. Therefore, understanding the microbial baseline and its responsiveness to dietary supplements like RPML in this specific breed is of particular importance. As a breed included in the National List for the Protection of Livestock Genetic Resources, and with increasing consumer demand for high-quality goat meat, optimizing the breeding efficiency of Leizhou goats, particularly by regulating their gut microbes to improve nutrient utilization, has become a focus of research [9,10]. Currently, nutritional studies on Leizhou goats mainly focus on the effects of tropical crop by-products on their growth performance and rumen fermentation. However, studies on the specific effects of RPML on Leizhou goats are scarce, and the potential mechanisms by which it affects their gut microbiota have not been fully elucidated.

Therefore, we hypothesized that dietary supplementation with RPML would improve the growth performance of Leizhou goats by modulating the hindgut microbial community and its metabolic output. By supplementing the diet with RPML, this work sought to determine its impact on the composition and function of the gut microbiota in Leizhou goats, which is of great theoretical and practical significance for improving the feeding management and breeding efficiency of Leizhou goats.

## 2. Materials and Methods

All experimental procedures involving animals were reviewed and approved by the Animal Care and Use Committee of the Zhanjiang Experimental Station, Chinese Academy of Tropical Agricultural Sciences (Protocol Approval Number: CATAS-20240005ZES).

### 2.1. Animals and Experimental Design

A total of 10 three-month old female Leizhou goats were randomly divided into 2 groups of 5 animals, which were designed as CON group (feed basal diet) and RPML group (added 1.5 g/d/head rumen-protected methionine + 4.5 g/d/head rumen-protected lysine to basal diet). The content of rumen-protected methionine and rumen-protected lysine was 50% and 56.8%, respectively, and the rumen passing rate was more than 85% (Kemin Industries, Incorporated, Des Moines, IA, USA). The experiment comprised a 14-day adaptation period followed by a 42-day formal feeding trial. All goats were housed under identical conditions, with feed provided twice daily at 09:00 and 16:00, and had free access to clean drinking water. They were fed a basal diet consisting of concentrate supplements and king grass as roughage. The concentrate was provided at a fixed amount of 200 g/day per goat, while king grass was offered ad libitum. The specific composition of nutritional requirements and nutrient content for Leizhou goats are shown in Table 1.

### 2.2. Sample Collection

Representative diet samples (approximately 100 g) were collected daily from day 36 to day 42 of the experimental period. At the end of the experiment, 10 goats were selected for sampling rectal fecal samples after a 12 h feed withdrawal period. Samples were immediately snap-frozen in liquid nitrogen and stored at −80 °C for subsequent analysis of short-chain fatty acids (SCFAs) and microbial community profiling.

### 2.3. Growth Performance Measurement

All goats were weighed individually after a 12 h feed withdrawal period on the 1st, 21st, and 42nd days of the feeding period. Cumulative feed intake was recorded to determine the average daily gain (ADG) and average daily feed intake (ADFI) during the experimental period. Dry matter intake (DMI) was calculated based on the records of the feed offered daily and the remaining feed weight the next day.

### 2.4. Feed Analysis

During the experimental period, a consistent weekly collection of 100 g of concentrate and an equal amount of king grass was undertaken. Subsequently, the samples were dried and subjected to analysis for dry matter (DM), crude protein (CP), ether extract (EE), neutral detergent fiber (NDF), and acid detergent fiber (ADF). The specific operation steps can be found in the report by Bahrampour et al. [11].

### 2.5. Assessment of SCFA Concentrations in Fecal Specimens

The concentrations of SCFAs in the fecal samples were determined by gas chromatography (GC). Frozen fecal samples were thawed at room temperature prior to analysis and subsequently homogenized thoroughly to ensure a representative mixture. The extract was obtained by dissolving 1 g of feces with 4 g of ultrapure water, followed by centrifugation at 15,000 rpm for 15 min at 4 °C. From the resulting supernatant, a 1000 μL aliquot was derivatized with 200 μL of metaphosphoric acid solution in an ice bath for 30 min. After a second centrifugation under identical conditions, the mixture was filtered through a 0.22 µm membrane. The processed supernatant was then injected into a Shimadzu GC-2010 plus system (Shimadzu Corporation, Kyoto, Japan) for analysis, which was fitted with a flame ionization detector and an AT-FFAP capillary column (30 m × 0.32 mm × 0.5 µm). The acetate-to-propionate (A:P) ratios were calculated based on their molar concentrations. Quantification was achieved by comparison against standard curves prepared for each SCFA.

### 2.6. DNA Extraction, Library Preparation, Sequencing, and Bioinformatics Analysis

A total of 10 individual fecal samples (5 per group) were collected under sterile conditions and preserved at −80 °C post-collection. Each sample was processed and sequenced individually; no pooling was performed. DNA was extracted using the QIAamp Fast DNA Stool Mini Kit (QIAGEN, Hilden, Germany), followed by quantification with a NanoDrop spectrophotometer. The V3–V4 region of the 16S rRNA gene was PCR-amplified with barcoded primers, and the resulting amplicons were purified, quantified, and sequenced on an Illumina MiSeq platform using a 2 × 300 bp paired-end strategy. Raw sequencing reads underwent quality filtering, joining, and demultiplexing within the QIIME2 pipeline. OTUs were clustered at 97% similarity, taxonomy was assigned using the SILVA database, and alpha/beta diversity metrics were computed. Statistical analyses, employing R software 4.4 and packages such as phyloseq 1.48, vegan 2.6, and DESeq2 1.44, identified differentially abundant taxa among study groups.

### 2.7. Statistical Analyses

The analysis of growth performance and fecal SCFA concentrations was conducted utilizing the SPSS version 23.0 software suite. Outcomes are reported as mean values accompanied by their standard error deviations (SEM). Statistical significance was defined as *p* < 0.05 based on t-test results.

## 3. Results

### 3.1. Effect of Dietary RPML on Growth Performance

The addition of RPML to the diet significantly improved the FBW and ADG of Leizhou goats (*p* < 0.001), as well as significantly decreasing the DMI (*p* = 0.023) and the ratio of DMI to ADG (*p* < 0.001) of the goats (Table 2).

### 3.2. Effect of Dietary RPML on SCFAs in Feces

As shown in Table 3, the concentrations of total SCFAs, acetate, and butyrate in the feces of RPML group were markedly lower than in the CON group (*p* < 0.05). The concentrations of propionate and Iso-SCFA, as well as the acetate-to-propionate ratio, showed no significant difference between the two groups (*p* > 0.05).

### 3.3. Summary of Combined Sequencing Datasets

We sequenced the 10 individual fecal samples (5 per group) via Illumina technology and produced a comprehensive set of 714,060 raw reads from 16S rRNA genes. Upon stringent quality control measures, a total of 449,830 reliable sequences emerged, which were subsequently grouped into 3761 distinct prokaryotic operational taxonomic units (OTUs), defined by a stringent 97% sequence similarity threshold.

**Figure 1:**
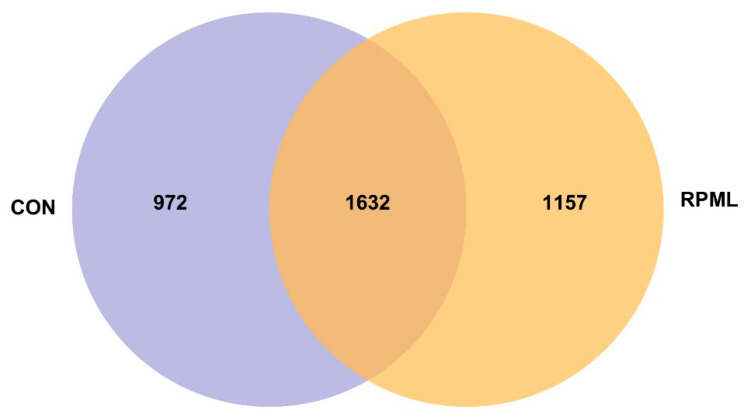
Venn diagram illustrating the distinct and shared operational taxonomic units (OTUs) in Leizhou goats fed with RPML. CON, control group and RPML, rumen-protected methionine and lysine.

As illustrated in Figure 1, this analysis revealed both distinct and shared OTUs in Leizhou goats administered with RPML. A significant overlap of 1632 OTUs was identified, representing 62.7% and 58.5% of the total OTU composition in the CON and RPML groups, respectively. Additionally, there were unique OTUs present in each group, with 972 exclusive OTUs identified in the CON group and 1157 in the RPML group.

Rarefaction analysis indicated that the sequencing depth was adequate to capture the abundance and diversity of the microbiota accurately. Despite the observed differences in OTU composition, statistical analyses showed no significant differences in diversity indices such as ACE, Chao, Coverage, Shannon, Simpson, and Sobs between the CON and RPML groups, as summarized in Table 4. The presence of a substantial number of unique OTUs in each group reflects the considerable individual variation inherent in the fecal microbiota. However, the large core of shared OTUs and the non-significant alpha diversity indices indicate that the overall community structure was stable and comparable, and the subsequent analyses focused on identifying statistically robust and biologically relevant changes within this variable background.

### 3.4. Effect of Dietary RPML on Fecal Microbiota Composition

**Figure 2:**
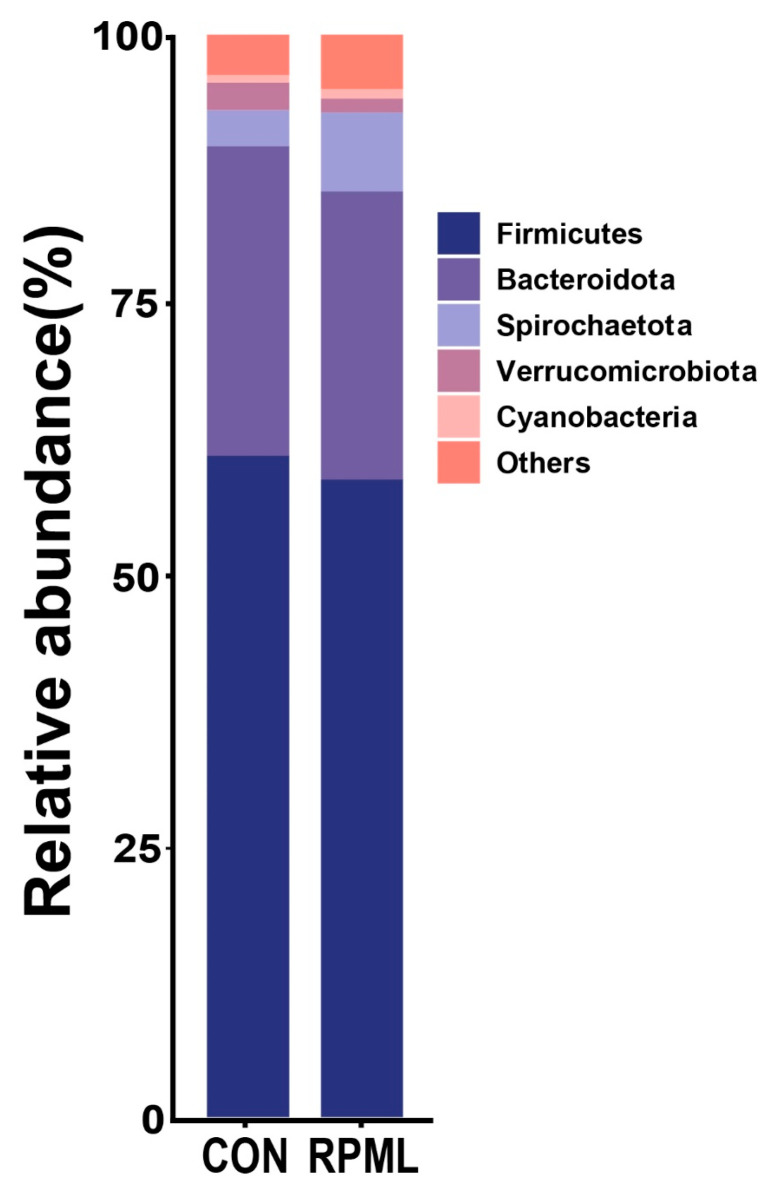
Fecal bacterial relative abundances (at phylum level, >1.0% of total reads) in Leizhou goats offered RPML. CON, control group and RPML, rumen-protected methionine and lysine.

At the phylum level, the results of OTU analysis of the fecal contents are shown in Figure 2. Among the 17 bacterial phyla identified in the two groups, Firmicutes and Bacteroidetes occupied the foremost and secondary positions, respectively, as the leading phyla in terms of abundance. The Firmicutes accounted for 61.11% and 60.54% in the CON group and RPML group, respectively, while Bacteroidetes accounted for 28.60% and 26.85%, respectively. The relative abundance of Verrucomicrobiota was remarkably decreased in the RPML group than in the CON group in Figure 2 (*p* < 0.05).

**Figure 3:**
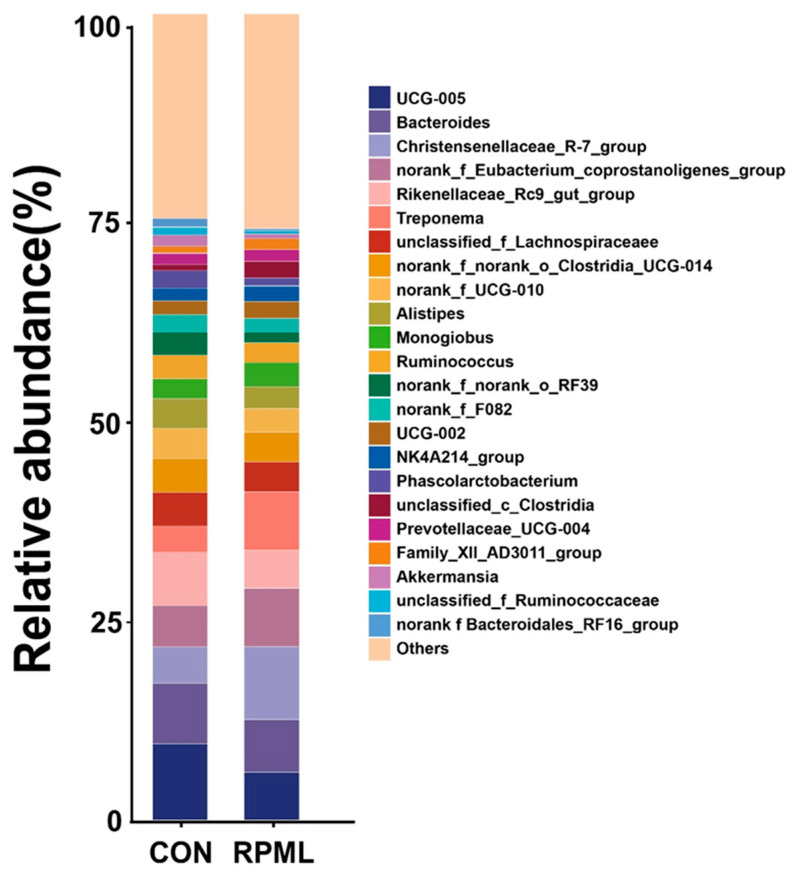
Fecal bacterial relative abundances (at genera level, >1.0% of total reads) in Leizhou goats offered rumen-protected RPML. CON, control group and RPML, rumen-protected methionine and lysine.

A total of 262 bacteria genera were identified from the analysis of all ten individual samples across the CON group and RPML group. The dominant genus was *UCG-005*, accounting for 9.46% and 5.95%, and the second-most-dominant genus was Bacteroides, accounting for 7.54% and 6.51% in the CON and RPML groups, respectively (Figure 3). The relative abundance of *Christensenellaceae_R-7_group*, *Treponema*, and *unclassified_c__Clostridia* were higher (*p* < 0.05) in the RPML group than in the CON group. The relative abundance of *UCG-005*, *Phascolarctobacterium*, and *norank_f__Bacteroidales_RF16_group* were lower (*p* < 0.05) in the RPML group than in the CON group.

**Figure 4:**
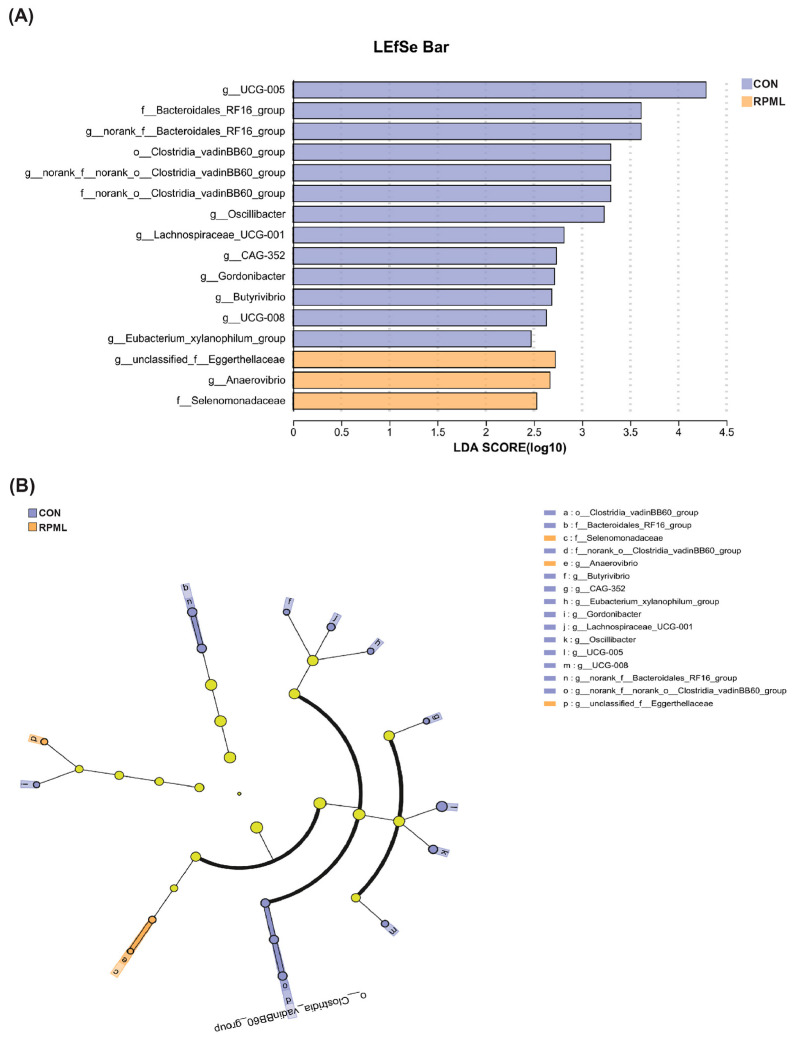
LEfSe results for fecal microbiota in Leizhou goats consuming diets including RPML. (**A**) Linear discriminant analysis. (**B**) Cladogram illustrating the phylogenetic relationship of the differentially abundant taxa. Key bacterial clades significantly enriched in the RPML group are highlighted with a yellow circle. Prefixes represent abbreviations for the taxonomic rank of each taxon: phylum (p_), class (c_), order(o_), family (f_), and genus (g_). CON, control group and RPML, rumen-protected methionine and lysine.

The disparities in the microbiota that fluctuated in response to RPML supplementation were more precisely delineated utilizing Linear Discriminant Analysis Effect Size (LEfSe) analysis, as depicted in Figure 4A,B. Applying the standard LDA threshold of ±2.0, we discerned 13 unique taxa within the CON group and 3 distinctive taxa within the RPF-supplemented group, respectively. The bacteria biomarkers in the CON group were *g_UCG-005*, *g_norank_f_Bacteroidales_RF16_group*, *g_norank_f_norank_o_Clostridia_vadinBB60_group,* and *g_Oscillibacter*, and in the RPML group they were *g_unclassified_f_Eggerthellaceae*, *g_Anaerovibrio*, and *f_Selenomonadaceae*.

### 3.5. Correlations Between Fecal Bacteria and Short-Chain Fatty Acids

**Figure 5:**
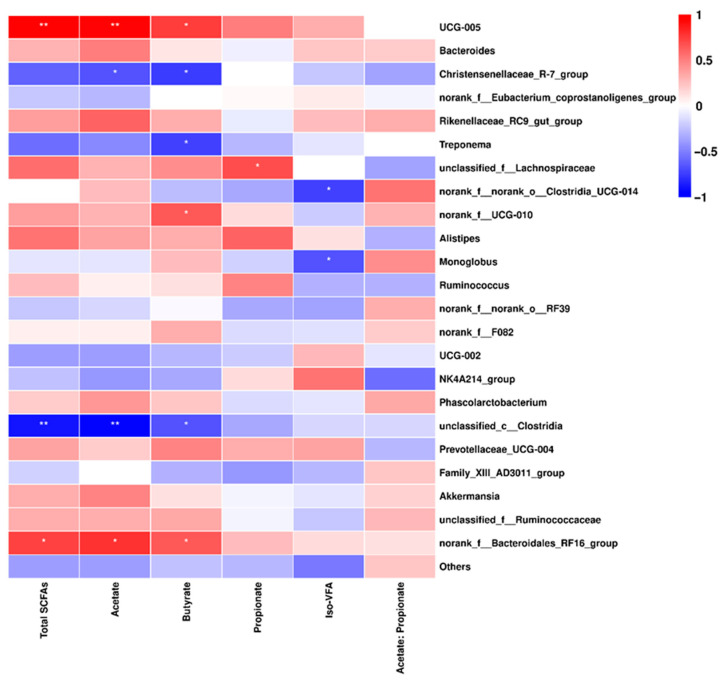
Correlation of fecal bacterial relative abundance at genus level with feces SCFA concentrations. * *p* < 0.05 and ** *p* < 0.01 according to the correlation coefficient.

A comparison of the CON group and RPML group showed a significant correlation between the relative abundance of bacterial genera and the concentration of SCFAs. Specifically, *UCG-005* and *norank_f__Bacteroidales_RF16_group* exhibited a positive correlation with total SCFAs, acetate, and butyrate, while displaying a negative correlation with propionate, Iso-SCFAs, and the ratio of acetate-to-propionate. Conversely, *Christensenellaceae_R-7_group*, *Treponema*, and *unclassified_c__Clostridia* presented opposite correlations (Figure 5).

## 4. Discussion

### 4.1. Diet with RPML Improves Growth Performance in Leizhou Goats

Optimizing ruminant diets to provide sufficient amino acids for growth, rather than excessive protein content, is an important strategy for improving growth performance, reducing nitrogen losses, and lowering feed costs. RPML, as an important limiting amino acid for ruminants, has a significant effect on growth performance. Here, goat diets supplemented with RPML significantly increased the ADG and FBW of Leizhou goats, in agreement with studies in calves [12], Holstein Bulls [13], and sheep [14]. Meanwhile, diets supplemented with RPML significantly reduced DMI and DMI: ADG compared to the CON group, and similar results were reported in yaks [15]. However, a lack of significant effect on growth performance was also reported by Wang et al. [16], which may be attributed to the supplementation of RPML during the close-up period of pregnant cows, where the primary metabolic focus is on gestation rather than postnatal growth. In our study, dietary RPML supplementation increased the ADG observed in the present study, effectively doubling that of the CON group, which underscores the critical nature of methionine and lysine as limiting amino acids in the basal diet for rapidly growing Leizhou goats. We posit that the basal diet created a specific deficiency in metabolizable methionine and lysine, which served as the primary constraint on protein accretion and growth. The supplementation of RPML directly relieved this constraint, leading to a more efficient utilization of dietary nitrogen for body tissue synthesis. This ‘unlocking’ effect can result in a particularly pronounced growth response, especially in young, growing animals with high protein turnover rates.

### 4.2. Fecal SCFA Production Altered by RPML Supplementation

In the present study, the addition of RPML to the diet significantly reduced the concentrations of total SCFAs, acetate, and butyrate in feces compared to the CON group. This reduction is unlikely to reflect diminished microbial fermentation but rather, may indicate enhanced absorption of SCFAs from the hindgut by the host [17]. As SCFAs represent a direct energy source for the host, contributing up to 10–20% of maintenance energy requirements in ruminants, their increased absorption could spare glucose and amino acids from being catabolized for energy. Consequently, more dietary and microbial amino acids, including the supplemented methionine and lysine, may be directed toward protein synthesis and muscle growth, thereby explaining the significantly improved ADG and feed efficiency in the RPML group [18,19]. This interpretation is consistent with the findings of Ferreira et al. [20] and provides a plausible mechanistic link between the modulated microbiota, SCFA dynamics, and the enhanced growth performance in our study.

### 4.3. Diet with RPML Influences Microbial Composition in Leizhou Goats

The abundance of the microflora reflects its ability to adapt to a particular environment and compete for available nutrients; moreover, it indicates its importance to the overall function of the microbiome as a whole [21]. This study investigated the effect of RPML on the fecal microbial community of Leizhou goats at the phylum level and genus level. The fecal flora of Leizhou goats was found to be dominated by Firmicutes and Bacteroidota at the phylum level, accounting for more than 80% of the total bacteria, which is consistent with the results of past studies on fecal microorganisms of ruminants [22]. Meanwhile, we found that the Verrucomicrobiota had a vivid reduction in the RPML group combined with CON group, which has the ability to promote intestinal barrier function and the physiological state of malnourished animal organisms [22,23]. Our research demonstrates that supplementing RPML in the diet increases nutritional intake in Leizhou goats, thereby altering fecal microbial community composition and ultimately improving growth performance.

Furthermore, the alpha diversity of the fecal microbiota was not affected between the two groups, indicating that the overall richness and evenness of the microbial community remained stable. However, this finding does not preclude specific compositional shifts. A well-established principle in microbial ecology is that community structure can be reconfigured while maintaining overall diversity [24]. Indeed, our results revealed that six bacteria were found to be significantly different at the genus level, with a decreased relative abundance of *UCG-005*, *Phascolarctobacterium*, and *norank_f__Bacteroidales_RF16_group* and increased *Christensenellaceae_R-7_group*, *Treponema*, and *unclassified_c__Clostridia* in the RPML-supplemented diet compared to the CON group. *UCG-005* has been reported in the literature to be positively associated with fiber fermentation and the production of SCFAs [25,26]. In the present study, the DMI was significantly lower in the RPML group, suggesting that the addition of RPML met the nutritional needs of the goats, leading to a voluntary reduction in roughage intake. This decrease in fibrous substrate passage likely altered the intestinal environment, resulting in a reduced abundance of fibrolytic genera such as *UCG-005*. *Phascolarctobacterium* is a genus of anaerobic bacteria belonging to the phylum Bacteroidetes and is closely associated with the production of SCFAs [27]. In the current study, the abundance of this genus was significantly lower in the RPML group compared to the CON group, which is consistent with results for fecal SCFAs [28]. In contrast, the present study showed that the ADG was significantly higher in the RPML group, suggesting that the RPML supplementation, which was responsible for the increased ADG, concurrently induced changes in the gut microbial community, including the decrease in the abundance of *Phascolarctobacterium*.

In addition, this study found that the abundance of *Christensenellaceae_R-7_group* was significantly increased in the RPML group. *Christensenellaceae_R-7_group* was previously reported to be significantly enriched in the hindgut of Hu sheep and to be effective in degrading fiber polysaccharides and producing beneficial metabolites in the colon [18]. In this study, RPML enabled goats to utilize dietary fiber polysaccharides more effectively to improve their growth by modifying gut microbes.

The observed alterations in the fecal microbiota following RPML supplementation are likely attributable to indirect mechanisms rather than the direct metabolism of rumen-protected amino acids in the hindgut. We propose that the enhanced amino acid profile absorbed in the small intestine improved systemic protein synthesis and host metabolism, which may have modified the secretory activity and intestinal milieu. These alterations subsequently changed the composition and availability of nutrients entering the large intestine, establishing a new ecological niche that favored the proliferation of specific bacterial genera such as *Christensenellaceae_R-7_group* and *Treponema*, while suppressing others including *UCG-005* and *Phaseolartobacterium*. This shift thereby contributed to the maintenance of gut health, promoted nutrient absorption, and enhanced productivity in Leizhou goats.

### 4.4. Dietary RPML Enhances Leizhou Goats Feed Efficiency by Modulating Gut Microbiota

As a bacterial family within the Firmicutes, *Christensenellaceae_R-7_group* was known to regulate gut homeostasis, thereby maintaining the health of goats [29]. In our research, the concentration of propionate in the feces shows a significant positive correlation with the abundance of *Christensenellaceae_R-7 group*. Although *Christensenellaceae_R-7_group* was enriched in the RPML group without a concurrent rise in fecal propionate, its positive correlation with feed efficiency suggests it may contribute to host energy metabolism through interactions with other gut microbes to stabilize community structure and enhance the degradation of complex polysaccharides, potentially leading to more efficient energy extraction [29]. Meanwhile, the diet with RPML supplementation significantly reduced the DMI: ADG in goats, indicating that the addition of RPML to the diet can alter the fecal content of *Christensenellaceae_R-7 group*, thereby improving feed efficiency in goats, which is in agreement with a previous study conducted in Angus Heifers [30].

## 5. Conclusions

In conclusion, dietary RPML revealed favorable influences on ADG and DMI. Moreover, some beneficial bacterial genera were significantly enriched after RPML addition such as *Christensenellaceae R-7 group*. Therefore, our results indicate that RPML supplementation is associated with altered gut microbiota composition and improved growth performance. The observed correlations between specific microbial shifts and SCFA profiles provide a plausible mechanism by which RPML may enhance nutrient utilization and feed efficiency in Leizhou goats.

## Figures and Tables

**Table 1:** Concentrate formulation and nutrient levels (dry matter basis).

Items	Content, %	Content of King Grass, %
Ingredients		
Corn	65.20	
Soybean meal	5.60	
Rapeseed meal	16.00	
Cottonseed meal	7.00	
Sodium bicarbonate	0.10	
Limeston	0.80	
NaCl	0.80	
Calcium hydrogen phosphate	0.50	
Premix ^1^	4.00	
Total	100.00	
Nutrient levels ^2^		
ME (MJ/kg)	10.66	
DM	95.25	93.56
CP	16.26	12.33
EE	11.54	9.98
OM	88.01	81.93
NDF	25.91	65.39
ADF	2.36	37.22
Calcium	0.57	
AP	0.10	

^1^ The premix supplied per kilogram of diet: Vitamin A, 7200 IU, Vitamin B1, 0.3 mg, Vitamin B2, 4.2 mg, Vitamin B5, 47 mg, Vitamin B6, 0.12 mg, Vitamin B12, 0.06 mg, Vitamin D3, 2060 IU, Vitamin E, 11 IU, Vitamin K, 30.05 mg. ^2^ ME, Calcium and AP are calculated. Other nutrient levels are measured. Abbreviations: ME, metabolic energy; DM, dry matter; CP, crude protein; EE, ether extract; NDF, neutral detergent fiber; ADF, acid detergent fiber; AP, available phosphorus.

**Table 2:** Effects of RPML addition on growth performance of Leizhou goats.

Items	CON	RPML	SEM	*p*-Value
IBW (kg)	9.87	9.95	0.08	0.622
FBW (kg)	10.80	11.80	0.17	<0.001
ADG (g/d)	22.22	44.05	3.53	<0.001
DMI (g/d)	258.50	236.02	5.26	0.023
DMI: ADG	11.76	5.36	1.06	<0.001

Abbreviations: CON, control group; RPML, rumen-protected methionine and lysine; IBW, initial body weight; FBW, final body weight; ADG, average daily gain; DMI, dry matter intake.

**Table 3:** Effects of RPML addition on feces concentrations of short-chain fatty acids in Leizhou goats.

Items	CON	RPML	SEM	*p*-Value
Total SCFAs, Mm	15.71	13.07	0.57	0.009
Acetate, Mm	12.28	10.49	0.41	0.016
Propionate, Mm	1.58	1.28	0.12	0.260
Butyrate, Mm	1.09	0.60	0.11	0.019
Iso-SCFA, Mm	0.77	0.69	0.03	0.212
A:P	8.47	8.28	0.62	0.887

Abbreviations: CON, control group; RPML, rumen-protected methionine and lysine; A:P, acetate: propionate.

**Table 4:** Alpha diversity indices in Leizhou goats feces as a reaction to RPML diet.

Items	CON	RPML	SEM	*p*-Value
Ace	1312	1355	55.12	0.719
Chao	1265	1305	50.07	0.709
Coverage	0.995	0.995	0.0002	0.962
Shannon	5.105	5.110	0.06	0.962
Simpson	0.016	0.016	0.001	0.921
Sobs	1160	1212	52.00	0.648

Abbreviations: CON, control group and RPML, rumen-protected methionine and lysine.

## Data Availability

The original contributions presented in this study are included in the article. Further inquiries can be directed to the corresponding authors.
